# Study of the ex vivo immune response of polytrauma older patients in the ICU on admission: preliminary results

**DOI:** 10.1186/cc13426

**Published:** 2014-03-17

**Authors:** L Filippou, K Venetsanou, G Voulalas, D Markopoulou, D Chroni, C Maltezos, I Alamanos

**Affiliations:** 1 KAT Hospital Athens, Kifisia, Athens, Greece

## Introduction

Immunological status is differentiated with age, influencing treatment and outcome [1] . The aim is to determine the immune response of severely traumatized older patients compared with a group with arterial disease, expressed by proinflammatory cytokine release after ex vivo whole-blood LPS stimulation [2] .

## Methods

The study comprised 16 polytrauma patients admitted to the ICU, aged 78 ± 8 (Group I) and 16 with arterial disease, aged 74 ± 5 (Group II). Ten milliliters of peripheral blood were collected from each patient, divided into two tubes with/without anticoagulant. Diluted 1:10 whole-blood samples were stimulated with 500 pg/ml LPS, at 37°C, for 4 hours. Serum and cell culture supernatants (CCSP) were removed and stored at -70°C. TNFα and IL-6 were measured in serum and CCSP by ELISA.

## Results

**Figure 1:**
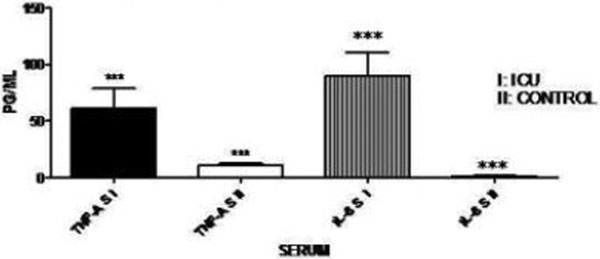
**Serum proinflammatory cytokines in older patients**.

**Figure 2:**
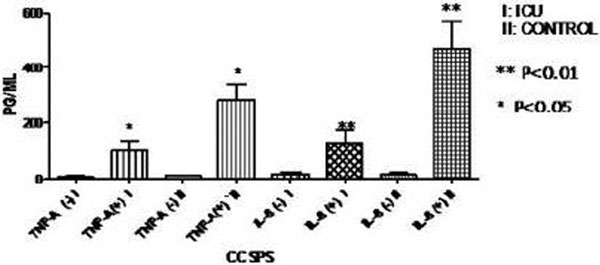
**Ex vivo proinflammatory cytokine release after whole-blood LPS stimulation in older patients**.

Serum proinflammatory cytokines were significantly elevated after severe trauma against control group (TNFα, *P *< 0.001 and IL-6, *P *< 0.001). Ex vivo cytokine release showed the opposite direction. There was a significantly lower TNFα and IL-6 release for Group I (TNFα, *P *< 0.05 and IL-6, *P *< 0.01) compared with Group II. TNFα ex vivo release from the samples of Group II was >300 pg/ml. See Figures 1 and 2.

## Conclusion

Older patients showed adequate immunological response, considering the limit of 300 pg/ml. The incidence of severe trauma was involved in the downregulation of immune activity and should be considered. Group I patients do not have the opportunity to precondition their immune status. Group II patients can better compensate operative therapies.
